# *In situ* cryo-EM reveals how a flagellar motor transmits high torque

**DOI:** 10.64898/2026.10.14.759264

**Published:** 2026-10-15

**Authors:** Jian Yue, Wangbiao Guo, Md Khalesur Rahman, Hui Xu, Md Samiul Haque, Aaron Ye, Huaxin Yu, Jun Liu, Md A. Motaleb

## Abstract

Bacterial flagellar motors are diverse rotary machines that require robust mechanical coupling to transmit torque across components with distinct architectures, yet how such coupling is achieved remains poorly understood. Here, we determine near-atomic *in situ* structures of the periplasmic flagellar motor in the Lyme disease spirochete *Borrelia burgdorferi* , revealing how an expanded rotor is mechanically coupled to the conserved rod through symmetry breaking and local remodeling of the rotor–rod interface. Surprisingly, pentameric FrbA forms a split washer-shaped brace around the rotor–rod junction, providing a second layer of mechanical reinforcement. Deletion of *frbA* destabilizes distal flagellar structures and impairs motility, particularly under increased viscous load. FrbA homologues are broadly distributed across spirochetes, suggesting a conserved mechanism for reinforcing high-torque flagellar motors. Together, these findings reveal how symmetry breaking couples an expanded rotor to a conserved driveshaft, while a molecular split washer reinforces this interface to enable robust torque transmission and motility.

